# The Inner Nuclear Membrane Has a Unique Lipid Signature

**DOI:** 10.1002/bies.70055

**Published:** 2025-08-18

**Authors:** Yang Niu, Tamas Balla

**Affiliations:** ^1^ International Center for Aging and Cancer Hainan Medical University Haikou Hainan China; ^2^ Section on Molecular Signal Transduction Eunice Kennedy Shriver National Institute of Child Health and Human Development National Institutes of Health Bethesda Maryland USA

**Keywords:** diacylglycerol, inner nuclear membrane (INM), lipid profiles, phosphatidic acid, phosphatidylserine

## Abstract

Although the inner nuclear membrane (INM) is generally considered to be continuous with the outer nuclear membrane (ONM) and connected to the remaining endoplasmic reticulum (ER), it has been well recognized that it is functionally distinct, having a unique protein composition. It has increasingly been recognized, however, that the INM also differs from the ONM and the other ER domains in its lipid composition. It is an intriguing proposition that the unique lipid profile of the INM is intricately linked to its specialized functions related to the nuclear events. Despite rapid progress in recent years in our understanding of the unique lipid profile of the INM and its role in the control of nuclear functions, there is a lot that remains to be understood. This review summarizes recent advances in characterizing the INM lipid composition and lipid synthetic pathways including their possible roles in the control of nuclear functions. Additionally, it discusses current challenges and areas deserving further investigation.

## Graphical abstract

The inner nuclear membrane (INM) has a distinct lipid profile, most notably characterized by high enrichment of phosphatidylserine (PS). In this review, Yang Niu and Tamas Balla summarize recent advances in elucidating the INM's lipid composition and related metabolic pathways, while elaborating further on their potential functional roles.

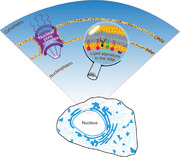

## 1 Introduction

All fundamental functions of eukaryotic cells depend on compartmentalization provided by organelle membranes, whose lipid composition also contributes to their unique signaling functions. The endoplasmic reticulum (ER) is the central organelle that synthesizes most of the cellular lipids [1, 2]. The synthesis of lipids in the ER not only impacts the structure and function of the ER itself [3, 4, 5, 6], but also functions as a hub that, together with the lipid transport processes, controls the lipid composition of membranes of other organelles [7, 8]. If any of these processes in the ER is derailed, it leads to diseases, such as obesity, insulin resistance, or hypertension, not to speak about diseases of the nervous system [9, 10, 11, 12, 13]. Understanding the importance of ER lipid sensing at a mechanistic level as it relates to lipid synthesis and distribution could help in identifying new therapeutic strategies.

**FIGURE 1:**
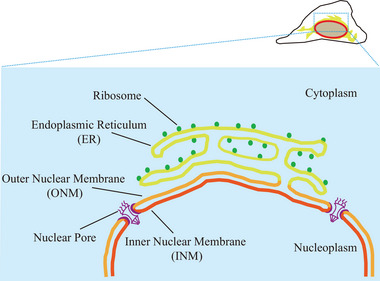
The relationship between the INM, ONM, and the ER proper. The nuclear envelope (NE) is a double‐membrane structure comprising the ONM and INM, separated by a luminal space, the nuclear pore complex, and the nuclear lamina in vertebrates (not shown). The ONM (orange) integrates seamlessly with the remaining bulk ER membrane (yellow), making it an extension of the ER network. In contrast, the INM (red), facing the nucleoplasm, connects to the ONM via the nuclear pore complexes (shown as purple structures), and maintains indirect connection with the rest of the ER (yellow), highlighting the structural interconnection of these membrane domains.

Several previous studies identified specific lipid sensing mechanisms by the ER, which contribute to the control of cellular lipid homeostasis. At the transcriptional level, the best known example is how cholesterol synthesis responds to exogenous cholesterol supply. Proteolytic activation of the transcription factors, SREBPs (sterol regulatory element‐binding proteins), in the cholesterol‐depleted state and its termination by cholesterol repletion have been worked out in a series of elegant studies [14, 15]. Other examples of transcriptional control include the response of cells to fatty acid loading or changes in nutrient supply [16, 17, 18, 19, 20, 21]. However, in addition to such transcriptional feedback regulation, the activity of lipid synthesizing enzymes can also be directly controlled by the lipid composition of the very membranes in which the enzymes reside. A good example is the negative feedback that phosphatidylserine (PS) exerts on the phosphatidylserine synthase 1 (PSS1) enzyme [22, 23, 24]. Breakdown of this feedback process as seen in gain‐of‐function mutations in the PSS1 enzyme causes Lenz‐Majewski syndrome (LSM) [11, 12]. With such a feedback control, lipid transport out of the ER becomes an important factor, because defective transport of lipids out of the ER to other organelles will immediately impact the ER lipid synthesizing machinery and ultimately affect the production of these lipids [25]. For example, PS is transported out of the ER to the plasma membrane (PM) by specific oxysterol binding protein related proteins (ORP 5/8) that use the concentration gradient of phosphatidylinositol 4‐phosphate (PI(4)P) between the PM and the ER [26, 27, 28]. Therefore, any process that affects the PI(4)P gradient will have an impact on PS synthesis [12]. A similar PI(4)P gradient‐driven cholesterol transport mechanism exists between the ER and the Golgi apparatus, which, again, tunes cholesterol synthesis to PI(4)P gradients [29, 30, 31, 32]. More comprehensive coverage of these regulatory processes can be found in several previous reviews [2, 33]. These examples were only described here to demonstrate the ability of ER to impact the cellular lipid composition of distinct organelles/subcellular membrane compartments via multiple mechanisms. It is an interesting question whether the inner nuclear membrane (INM), which is connected to the outer nuclear membrane (ONM) via nuclear pore complexes and, therefore, indirectly linked to the ER proper, as shown in Figure 1, plays any role in these regulatory processes.

It is a generally accepted view that the unique lipid composition of distinct organelle membranes (e.g., PM, endosomes, ER and mitochondria) contributes to the unique function of these organelles by defining their signaling properties [34, 35]. These properties include, but are not limited to, lipid‐mediated protein recruitment, determining protein conformation and protein assembly dynamics, or defining channel gating properties. For example, phosphatidylinositol phosphates in the PM or endosomes serve as docking sites for proteins that contain pleckstrin homology (PH), Phox homology (PX), or FYVE domains, or help assemble multimeric protein complexes that control complex cellular processes such as the starvation response [36, 37]. Given the fact that the INM faces the nucleoplasm, it is not unreasonable to assume that the lipid composition of the INM may differ from the rest of the ER, to suit the specific signaling functions communicated toward the intranuclear environment. The INM proteome, first characterized by Schirmer et al. [38], clearly shows a unique protein makeup of the INM as reviewed in [39, 40]. However, studies that would complement the distinct INM proteome with the INM lipidome are generally lacking. For a while, the general prevailing view has been that the lipid composition of the INM is not particularly different from those of other regions of the ER, including the ONM. Direct testing of this question by precisely determining INM lipid composition has proven technically challenging. Methods employed to address this include biochemical isolation of nuclear envelopes followed by the lipidomic analyses. Despite considerable efforts to improve these isolation techniques, this approach still suffers limitations, such as the potential contamination from the ONM or peripheral ER [41]. Moreover, organelle fractionation is inherently disruptive and may result in damaged organelles, and potentially altered lipid composition [42]. Therefore, there is a growing need for alternative, preferably in situ detection strategies, such as live cell tracing. As most of the water‐soluble ingredients used for lipid synthesis are found in the cytoplasm and not in the nucleoplasm, lipid synthesis is believed to be primarily associated within the ER proper, away from the INM. Consequently, newly synthesized lipids were thought to simply diffuse laterally throughout the ER membranes, reaching the ONM and then the INM through membrane junctions that are part of the nuclear pores. Several recent studies suggest that these views may have to be revisited, as unique enrichment of some of the lipid synthesizing machineries was specifically found in the INM and the INMs were shown to have lipid composition that differs from that of the ER proper [17, 43, 44, 45].

In this review, our aim is to draw attention to nuclear lipids and focus on lipids that are present in the INM. It is important to examine both the processes by which these lipids are produced and their possible functions within the nucleus. We will also highlight areas where nuclear functions controlled by the INM through its unique lipid metabolism could benefit from further investigation.

## 2 Lipid Synthetic Machinery in INM

Studies on lipid synthesis and lipid fluxes in the context of the INM in eukaryotic cells greatly benefited from research in yeast, due to the ease of genetic manipulations. The next section describes both common and unique features of lipid classes that are found enriched in the INM, including the specific enzymes that are involved in these metabolic processes and are conserved from yeast to higher metazoan organisms.

### 2.1 The “Phosphatidic Acid ⇆ Diacylglycerol” Balance

**FIGURE 2:**
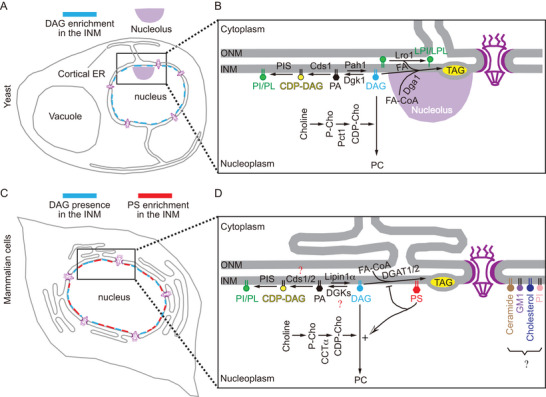
Lipid classes and related pathways in the INM in yeast and mammalian cells. (A) The enrichment of DAG in the INM in yeast. (B) The lipid composition of the INM is defined by the “PA ⇆ DAG” balance in yeast: specifically, the DAG produced in the INM can be used for either PI/PL synthesis through the Pah1‐Cds1‐PIS enzymatic cascade; or for PC synthesis through the CDP‐choline pathway. Additionally, DAG can be used for TAG synthesis through the Dga1 or Lro1 pathways. Cell cycle delay or arrest, and nutrient starvation induce the relocation of Lro1 protein from the ER to INM [79], where it transfers the acyl‐groups from the sn‐2 position of phospholipids to the acceptor DAG molecule in the INM, thereby enabling the synthesis of TAG and the concomitant production of lyso‐phospholipids [112, 113]. (C and D) The prominent presence of DAG and the enrichment of PS in the INM found in the mammalian cells. PS found in the INM promotes the channeling of the DAG to PC synthesis through the CDP‐choline pathway as it helps recruit critical enzymes for PC synthesis to the INM. More studies would be warranted to explore the distribution of DGKs, Cds1/2 enzymes and the possible roles of ceramide/GM1/cholesterol/phosphatidylinositol (PI) in nuclear functions (as indicated by the black question mark).

This was one of the best studied lipid conversion pathways that was shown to also operate within the INM. Tipping this balance toward the production of PA promotes phospholipid synthesis, whereas tipping it toward diacylglycerol (DAG) formation leads to either phosphatidylcholine (PC) synthesis via the CDP‐choline branch of the Kennedy pathway or lipid storage (see Figure 2B,D). DAG, one of the two core lipids in this equation, has been detected in the INM both in yeast [17] and in U2OS cells [43], with a suggested higher concentration in the INM than in other domains of the ER shown in yeast [17]. Although the prominent presence of DAG in the INM has been clearly demonstrated in the U2OS cells [43] and DAG has been also detected in the ER [46, 47], because of the lack of systematic comparisons, it is not certain whether DAG is also enriched in the INM in mammalian cells. It may be important to point out that DAG biosensors based on C1a or C1a‐C1b domains isolated from different proteins may respond differently to DAG pools depending on the fatty acyl side chain composition of DAG species [48]. On the basis of these distinctions, it was suggested that polyunsaturated fatty acyl (PUFA)‐containing DAG species are present in the INM of U2OS cells [43]. PA, on the other hand, cannot be detected in the INM at steady state, most likely due to its low amount that is not sufficient to be detected by biosensors against the unbound high background signal [17, 49]. The Q2 domain of wildtype Opi1, however, was able to detect higher PA levels in the INM after depletion of the PA phosphatase, Pah1, or after overexpression of Dgk1 in yeast [17]. A recently developed enhanced PA sensor, based on the tandem Q2 domain of Opi1 with a mutation that boosts its PA‐binding affinity, however, could detect even the low‐level PA in the INM in *Schizosaccharomyces pombe* and follow the changes in both DAG and PA levels during mitosis and expansion of the NE membrane [44]. Using this PA biosensor in yeast, Seipin was found to be an important factor determining the level and fate of PA in the INM [50]. It would be of great interest to test this biosensor in mammalian cells to see whether the same mechanism of turning lipids to build membranes is evolutionally conserved.

Additional support to associate the DAG–PA balance with membranes of the nucleus is provided by the presence of the key enzymes that determine this lipid balance in the NE membrane. The rate‐limiting enzyme in the production of DAG from PA in yeast is the Mg^2+^‐dependent PA phosphatase enzyme, Pah1 (also termed SMP2) first identified decades ago [51, 52]. Pah1 is the substrate of the Nem1p‐Spo7p complex, which dephosphorylates Pah1, thereby increasing its enzymatic activity [51, 53, 54]. Barbosa et al. showed localization of active Pah1 with the nucleo‐vesicular junction (NVJ), where its activity determined the balance between lipid storage and membrane biogenesis, providing the first insights into the compartmentalized Pah1‐mediated lipid flux at the NE [55]. Romanauska and Köhler further narrowed the precise location of Pah1 to the nucleoplasmic side of the NE, where catalytically active Pah1 can access PA within the INM and direct DAG product to TAG and formation of nuclear LDs (nLDs) [17]. The INM localization of the mouse and human homologs of yeast Pah1, Lipin1 (Lipin1α and Lipin1β), have also been observed when activated by various factors in multiple studies. For example, Lipin1α (the variant showing highest nuclear localization) is recruited to INM in response to oleic acid (OA) exposure [45, 56, 57, 58, 59, 60, 61, 62]. Furthermore, CTD Nuclear Envelope Phosphatase 1 (CTDNEP1 or CNEP‐1 in *Caenorhabditis elegans*, also known as Dullard) and its associated NEP1‐R1, which are homologs of the yeast Nem1p‐Spo7p phosphatase complex that regulates Lipin1 activity, have been shown to be enriched in the NE [63, 64, 65, 66], and also present in the INM [43]. Another enzyme, CCTα (CTP:phosphocholine cytidylyltransferase alpha) that generates CDP‐choline for PC synthesis, is also prominently present in the nucleus and associates with the INM upon OA loading [45, 67] (and see below).

DAG can have different fates, likely depending on where it was produced. DAG can be converted back to PA by DAG kinases (DGKs), and PA can serve as a substrate for CDP‐DAG synthase (CDS) enzymes for synthesis of phosphatidylinositol (PI) or phosphatidylglycerol (PG). In yeast, both Dgk1 and Cds1 are present in both the INM and ONM, as evidenced by immune‐electron microscopy (EM) [17]. In mammalian cells, 10 different DGKs and 2 CDS enzymes are present [68, 69]. Mammalian Cds1/2 are located primarily in the ER; their INM localization as well as those of DGKs remain to be elucidated (indicated in Figure 2D by a red question mark). DAG can also be used to produce PC through the Kennedy pathway. The rate‐limiting step in this reaction sequence is the production of CDP‐choline from phosphocholine by the CCTα enzyme [70]. As mentioned above, the CCTα protein is localized in the nucleoplasm and is recruited to the INM upon demand. The CCTα homolog, Pct1 in yeast also localizes to the nucleus [71, 72]. Although Pct1 may also be present outside of the nucleus associated with the ONM, Pct1 in the nucleoplasm can have immediate access to the INM. The lack of Pct1 in the peripheral ER membrane has also been shown [55]. The nuclear localization of mammalian CCTα has been more solidly proven by several means including immuno‐EM showing its presence at the INM and nuclear reticulum (NR) [73]. Translocation of CCTα to the INM and NR represents an important step in the synthesis of PC and has been shown to contribute to for the expansion of the NR [73, 74]. While the formation of NR may not exclusively depend on CCTα‐mediated synthesis of PC [75], the expansion of NR may help to alleviate the burden of excess FA through synthesis of phospholipids when OA loading occurs. An additional PC synthetic route, the phosphatidylethanolamine (PE) methylation pathway exists in the ER in yeast and in some mammalian cells, but its link to the nucleus has yet to be explored [76, 77]. Lastly, DAG generated in the INM can be used to synthesize TAG for lipid storage. The activity of the diacylglycerol‐acyl transferase (DGAT) enzymes required for TAG synthesis, such as Dga1 and Lro1 in yeast, and DGAT1/2 in mammalian cells, must be at least partially present in the nucleus as formation of nLDs has now been well documented [78, 79].

### 2.2 Phosphatidylserine Synthesis and Nuclear PS

In a recent study [45], using various approaches including the use of PS biosensors Lact^C2^ or Evt^2xPH^ that have been tested in a variety of different studies [80, 81, 82], PS was found enriched in the INM in live mammalian cells, including U2OS, HeLa, Huh7, and primary mouse cortical neurons (also see the cartoon in Figure 2C,D). Taking advantage of the different PS affinities of these two PS biosensors (Lact^C2^ and Evt^2xPH^), these studies found that PS is asymmetrically distributed between the two leaflets of the NE membranes. The leaflet of the ONM facing the cytoplasm had the lowest PS content, whereas the nucleoplasmic face of the INM exhibited the highest PS content and the ER luminal aspect of the NE membrane falls in between. Importantly, even the highest PS density of the inner leaflet of the INM was below that of the inner leaflet of the PM. These findings assumed that an active mechanism exists that maintains such an asymmetric PS distribution. This PS distribution was in agreement with earlier observations [82, 83], but not with an EM study that used lipid (PS) detection on freeze‐fractured replicas. The EM study found that the ER cytoplasmic leaflet has a PS content as high of as the inner leaflet of the PM [84], an assertion that is not supported by live cell imaging studies [45, 85]. Whether this discrepancy is due to the differences between sample preparations for EM vs. light microscopy, or to the inability of the PS probes to access a large PS pool in the cytoplasmic leaflet of the ER in live cells, will have to be clarified in future studies. As the nuclear membrane proteome is significantly different between cell types [86], one cannot rule out potential differences in the PS distribution among the different cell types including MEF cells used in the EM studies. As for functional relevance of the INM‐enriched PS, membrane recruitment of two key enzymes for PC synthesis, CCTα and Lipin1α, was shown to require nuclear PS or perhaps other negatively charged lipids (see below).

### 2.3 Other Lipids Detected in the INM

In addition to the lipids discussed above, other lipids have been shown to be present and play important roles in the INM in metazoan organisms. One important pathway that was featured in the nucleus is the cytosolic phospholipase A2 (cPLA_2_) pathway yielding arachidonic acid (AA) for the formation of cyclo‐oxygenase and lipoxygenase products. It was observed that a physical injury in zebrafish tail fin induces a wound‐healing response with leukocyte activation that is triggered by cPLA_2_ activation in the nucleus [87]. Here the trigger appears to be the mechanosensitive reaction of the nucleus to osmotic swelling [88]. A similar nuclear cPLA_2_ and 5‐lipoxygenase‐mediated leukotriene formation was found in neutrophils induced locally by neutral sphingomyelinase‐1, which generates ceramide‐enriched lipid‐ordered microdomains in the NE [89]. It is also noteworthy that the Lamin B receptor (LBR), which is resident in the INM, has been found to play a role in providing cholesterol presumably to the INM through its sterol C14 reductase activity [90, 91]. Another sterol C14 reductase, TM7SF2, is found in the ER proper; therefore, it is not clear which nuclear membrane compartment has a higher cholesterol content or more importantly, whether the roles of the two enzymes are distinct or complementary. Two early studies reported that cholesterol is more likely to be present in the ONM than in the INM based on filipin‐staining [92, 93], although filipin is not the most sensitive method to detect cholesterol distribution. More studies are warranted to explore cholesterol or sphingolipid biology in the context of nuclear membrane domains (as indicated in Figure 2D with a black question mark).

Another lipid class that has attracted lot of attention in the nucleus is the phosphoinositides. Several studies found inositol phospholipids or their metabolizing enzymes within the nucleus, and it was shown that some of these lipids are not membrane associated, but are found to be bound to transcription factors [94, 95] or being covalently attached to proteins such as p53 [96, 97]. These exciting developments in nuclear phosphoinositides have been covered in excellent reviews and will not be discussed here in further detail [98, 99, 100, 101].

## 3 How Does Lipid Composition Affect INM Functions?

The unique lipid composition of the INM and its enrichment in several lipid‐synthesizing or ‐modifying enzymes have raised the question of their functional significance. The following section will discuss some of the identified functions:

### 3.1 Regulating Protein Localization to the INM

The unique lipid composition of organelle membranes determines their biophysical properties, such as their surface charge, packing defects, and curvatures as reviewed in [6]. However, beyond biophysical properties, lipid composition plays a big role in determining what proteins are associated with these membranes. In the case of the INM, the enrichment of lipids such as PS, DAG, and the presence of PA even in low amounts, together with the other structural lipids, PC and PE, not only determine its unique biophysical properties, but also define the protein makeup of the INM. It has been shown that amphipathic helices (AHs) present in nuclear proteins play a uniquely important role in their INM localization [102]. This was exemplified by the nuclear protein, Sun2, whose DAG‐dependent lipid binding to the INM controls its proteolytic degradation [43]. AHs can sense DAG‐rich membranes or packing defects, and most likely many other proteins that associate with the INM use this mechanism [102].

**FIGURE 3:**
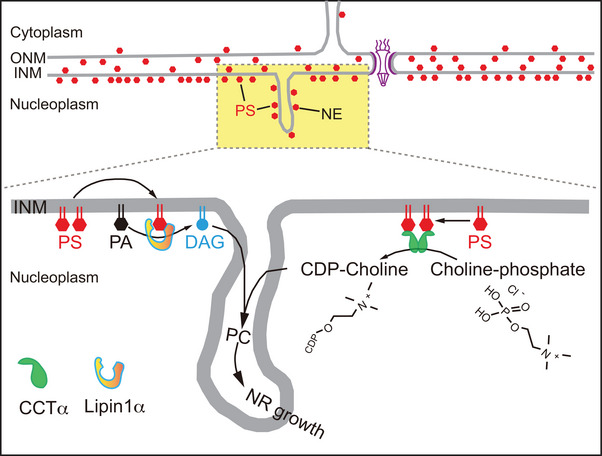
Recently described asymmetric distribution of PS across different leaflets of the NE in mammalian cells: the cytosolic leaflet of the ONM, the luminal leaflets of both ONM and INM, and the nucleoplasmic leaflet of the INM. Notably, the enrichment of PS in the INM in mammalian cells can drive PC synthesis via membrane recruitment of CCTα and Lipin1α in response to oleic acid loading.

Electrostatic interactions mediated by negatively charged lipids (e.g., PA, PS) are also important for INM association of proteins. For example, it has been shown that the repair of INM damage requires the recruitment of the specific ESCRT protein, Chm7 by local PA enrichments in the INM [103]. We have already mentioned that PS enrichment in the INM, together with AH–lipid interaction [104], is an important contributor to the membrane recruitment of CCTα upon loading of cells with OA [45]. Similarly, Lipin1α was found to require the C terminal segment of its M‐Lip domain to bind to the INM in cells loaded with OA [45]. Accordingly, in vitro binding experiments have shown that the M‐Lip domain of Lipin1α interacts with both PS and PA [105], suggesting that the interaction may not be specific to a particular lipid class, but rather to negatively charged lipids in general. However, when nuclear PS was depleted in intact cells, the translocation of both CCTα and Lipin1α to the INM was impaired, which would be consistent with the fact that PS is the main negatively charged lipid providing the electrostatics in the INM (see Figure 3). As discussed above, in addition to the membrane electrostatics, membrane packing defect and curvature, are both critical for the membrane recruitment of CCTα. The M domain of CCTα has been shown to sense the packing defects and curvature strain caused by reduced PC levels and PC/PE ratio [106].

When it comes to protein membrane association, the relative importance of packing defects and curvature also depends on the physical properties of the lipids that make up the membrane, including the INM. The larger head group of PC makes it form a cylindrical shape, which lessens packing defects and curvature strain. Importantly, PS is also of cylindrical shape in addition to being anionic. In contrast, PE, with a smaller head group, assumes a conical shape that increases packing defects and promotes negative curvature strain. PA and DAG also belong to the group of conical shaped lipids; therefore, besides PC level and PC/PE ratio, the contribution of these other lipids must be considered in relation to packing defects and curvature.

### 3.2 Nuclear Pore Function

In addition to localization of proteins to the INM, an additional and poorly explored area is the potential regulation of the gateway function of the nuclear pore complex (NPC) by the lipid composition of the nuclear membrane. This regulation involves two key aspects of the NPC structure. First, NPC assembly must adapt to the highly curved membrane at NPC sites, where the ONM and INM are connected with a membrane of high curvature. And the lipid composition at these loci must accommodate this high membrane curvatures. Proteins that form the NPC also need to assemble and, in some part, penetrate these highly curved lipid domains. This penetration process critically depends on the local lipid composition where fatty acid side chain saturation has been shown to play an important role [107]. On top of their simple assembly, the constricted and dilated states of the NPC have been shown to rely on the mechanosensitivity of these structures, which likely involves lipid remodeling [108]. These molecular states ultimately determine the barrier function of the NE that is thought to be influenced by the local lipid composition within the INM [65, 108]. Moreover, packing defects and membrane curvature, determined by the ratio of lipids with varying shapes, are sensed by nuclear pore proteins with AHs or ALPS domains, such as those found in Nup133 [109, 110]. Further details on this topic can be found in a recent review [111].

### 3.3 Balancing Membrane Expansion, NE Integrity, and Lipid Storage

As pointed out above, DAG serves as a metabolic hub in the INM. It can be used for lipid storage in the form of lipid droplets or consumed for phospholipid synthesis via either the Kennedy pathway (PC, PE and PS in yeast) or PI synthesis after conversion of DAG to PA and then to CDP‐DAG. In the fission yeast *S. pombe*, during the process of closed mitosis, NE membrane expansion follows a well coordinated process that is a prerequisite for the formation of two daughter nuclei. Here, directing DAG flux toward phospholipid synthesis in the INM provides the lipid source for the NE membrane expansion [44]. In the budding yeast, *Saccharomyces cerevisiae*, the phospholipid diacylglycerol acyl‐transferase enzyme, Lro1, is located in the INM in close proximity to the nucleolus to facilitate TAG synthesis, which is important to maintain the integrity of NE [79, 112, 113, 114, 115]. In metazoans, such as in *C. elegans*, during open mitosis, DAG formed in the INM by the Lipin1 homolog, *lpin‐1* gene (H37A05.1), controls NE dynamics [116], including NE breakdown during mitosis [66, 117]. It was suggested that nuclear DAG activates PKC enzymes situated in the nucleus to initiate the phosphorylation and subsequent depolymerization of the Lamin (LMN‐1) protein that is essential for NE breakdown [117]. This was corroborated in subsequent studies performed in HeLa cells [118]. Additionally, two earlier studies suggested that a rapid rise in nuclear DAG occurs at the transition of the G2‐M phase in mammalian cells [119, 120]. DAG depletion was also shown to interfere with NE reformation after mitosis [121]. Another study in *C. elegans* suggested that NE breakdown is associated with reduced PI synthesis due to the conversion of PA to DAG by Lipin1, the latter activated by CNEP‐1, the *C. elegans* homolog of CTDNEP1 [64]. In the same study, a shift to increased PI synthesis was shown to lead to expansion of ER sheets and slowing NE disassembly during cell division. Thus, redirection of lipid metabolism toward DAG synthesis away from PI during mitosis is critical to prevent excessive ER sheet formation near the NE and to ensure proper NE disassembly [66]. These abundant ectopic ER sheets may stabilize the NE, but could also physically interfere with nuclear pore function, potentially hindering the nuclear translocation of key mitotic regulators such as the cyclin‐B1–Cdk1 complex [122]. It is noteworthy that a recent high‐content screen also identified CTDNEP1 as an important regulator of the repair of nuclear membrane rupture [123], likely through modulation of nuclear membrane quantity and lipid composition through Lipin1 activation.

### 3.4 Gene Regulation

**FIGURE 4:**
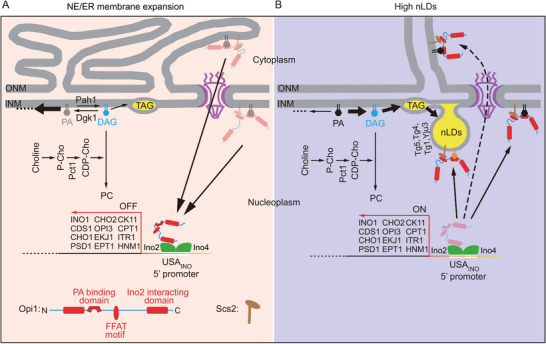
Lipid levels in the INM regulate gene expression in yeast. (A) When cells undergo NE/ER membrane expansion, PA levels are dropped due to increased consumption (indicated by the lightly black PA), which leads to liberation of Opi1 from the membranes and repression of phospholipid biosynthesis to limit excessive membrane expansion. (B) Under conditions when DAG is channeled toward lipid droplet (LD) formation, Opi1 would relocate to the surface of nLDs, the INM and ER, and phospholipid biosynthesis is de‐repressed. The lightly colored versions of Opi1 indicate the process of Opi1 dissociation from its binding sites in both (A) and (B).

One of the best characterized lipid‐controlled gene regulations is known in *S. cerevisiae*, where Opi1 acts as a repressor of several phospholipid biosynthetic genes. In its inactive form, Opi1 localizes to the ER with a slight enrichment in membranes of the NE (i.e., INM, ONM) [21] and in the single phospholipid layer that encapsulates lipid droplets (LDs) (both cytosolic [cLDs] and nuclear [nLDs]) [17]. This membrane association of Opi1 in the ER is mediated by specific interaction with PA together with the integral ER protein, Scs2 [21, 124]. When the PA levels drop, Opi1 is released from the membranes and is readily translocated to the nucleoplasm, where it binds to the UAS_INO_ element within the promoter regions to suppress the transcription of phospholipid biosynthetic genes (see Figure 4A). Consequently, it is the “PA ⇆ DAG” balance within the INM that controls expression of phospholipid synthesizing genes and, hence, fine tuning the lipid flux toward either phospholipid biosynthesis or lipid storage. Consumption of PA during various metabolic conditions, such as during the addition of myo‐inositol, can then be the ultimate signal to control the expression of lipid synthetic genes [16, 125, 126, 127]. An additional example of how lipid changes in the INM could regulate gene expression is through the formation of nLDs. Enrichment of PA on the surface of nLDs was observed, which could draw Opi1 away from the UAS_INO_ domain to de‐repress the genes for phospholipid biosynthesis [16, 17] (see Figure 4B). So, there are two different scenarios of gene control, which suit the formation of phospholipids: one that is associated with ER membrane expansion, and one that ensures that growing nLDs will have sufficient phospholipids to form their single layer lipid coats (see Figure 4).

**FIGURE 5:**
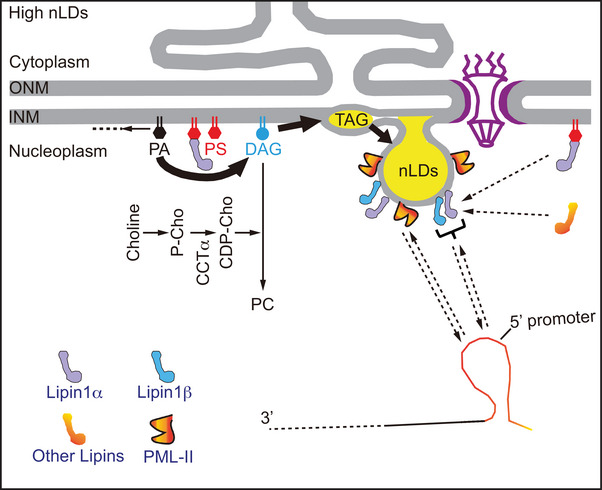
Nuclear LDs serve as docking sites for Lipin1 (both α and β) and nuclear bodies scaffolded by PML‐II. How this association controls gene expression remains to be determined. (Arrows with dotted lines indicate the processes are uncertain and represent research areas that remain to be explored).

It is also important to point out that Lipin1 and 2 also have transcriptional activities and, through interaction with transcription factors, can control the expression of genes important for lipid metabolism [128, 129, 130] (reviewed in [19]) (see Figure 5). Another example of gene regulation is by the Promyelocytic Leukemia Protein II (PML‐II), which serves as a scaffold of PML nuclear bodies. Upon oleate loading, PML bodies and nLDs recruit the CCTα and Lipin1 enzymes [18]. Overexpression of PML‐II also upregulates nLDs [18, 131, 132]. Chromatin interactions with nuclear bodies are known to be important to regulate genome functions [20], but how their association with nLDs and the PC synthesizing enzymes affects these functions remains to be determined (Figure 5).

It is currently unclear whether additional proteins exist that specifically interact with lipids, such as PA and PS, in the INM, but studies are already in progress that use proximity labeling methods to identify nuclear proteins whose activity is linked to lipid changes such as those induced by OA loading [133].

## 4 How Do Lipids Get Enriched in the INM?

The most obvious mechanism for creating and maintaining the enrichment of specific lipids in the INM is their synthesis in situ in the INM or its subdomains. As discussed above, good examples are the localization of the yeast Pah1 and its activator Spo20/Nem1 complex in the INM producing DAG at this location. This is an evolutionarily conserved feature, which is also found in mammalian cells [134, 135]. It is a more complicated question of how lipids that are primarily synthesized in the ONM, the ER proper, or even in other organelles such as the Golgi can find their way to the INM, let alone being enriched there. Lipids synthesized in the cytoplasmic surface of the ER can use scramblases to distribute equally in the luminal and cytoplasmic leaflet of the ER, but would require a flippase to establish an asymmetric PS distribution along the ER or membranes of the NE [136]. Naturally, lipids could diffuse laterally and equilibrate between the ONM and INM at NPCs, although whether these sites represent lipid conduits or barriers between these membrane domains remains to be seen (see more on the possible barrier function below). Our recent report showing PS enrichment in the INM relative to the ER lumen and the cytoplasmic face of the ONM and the ER proper makes this question even more relevant. PS transport mechanisms have been reviewed recently in detail [137, 138]. As mentioned above, EM studies found asymmetric distribution of PS in the ER proper, presumably established by P4‐ATPases, as it is well established for the PS enrichment of the cytoplasmic leaflet of the PM. In that nuclear study, the TMEM16K scramblase was shown to induce a Ca^2+^‐mediated increase in the PS content of the nucleoplasmic face of the INM [84] and it was suggested that even the barrier function of the NPC might be affected by the Ca^2+^ rise [139]. It is important to point out that the PS level in the INM very closely mirrored the activity of the PSS1 (and possibly PSS2) enzymes that are mostly located in the ONM and ER proper [45]. Efforts to identify the P4‐ATPase(s) or scramblases that are responsible for the INM PS enrichment have been unsuccessful, likely because of redundancy in the system and more studies will be required to understand this process [45].

Differences between the lipid composition of the ER, the ONM, and the INM suggest that the lateral diffusion of lipids in the membranes between the INM, ONM, and the remainder of the ER is limited and that barriers exist that limit the free lateral diffusion of lipids between these membrane domains. A recent study has shown in fission yeast that the Lap2‐Emerin‐Man1 (LEM) domain protein Lem2, located in the INM, serves as a barrier to restrict membrane flow into and out of the INM and another protein, Lnp1, located in the ER, acts as a secondary barrier limiting membrane flow between the NE and ER proper [140]. Notably, double knockout of Lem2 and Lnp1 also leads to the leakage of proteins out of the nucleus pointing to a major organizational breakdown of the NE [141]. Defects of these barrier functions will inevitably lead to loss of any lipid enrichment that exists in the INM, a prediction that needs experimental confirmation. Adding to its possible regulatory roles, Lem2 interacts with lipid metabolic enzymes (Cho2, Ole1, Erg11) and, as such, is involved in phospholipid and sterol biosynthesis, whereas its partner Bqt4 binds Cwh43, an enzyme involved in lipid remodeling during GPI anchor maturation [142]. Therefore, Lem2 may not only restrict lipid diffusion but also participate in local lipid production to sustain INM enrichment. It will be important to determine if a similar barrier mechanism is functional in mammalian cells and if so, to elucidate the mechanism involved. A physical structure termed “constricted junction” has recently been reported in mammalian cells (but not in budding yeast) to regulate ER‐to‐NE lipid and protein diffusion in higher eukaryotes. However, the molecular composition in the “constricted junction” is still unknown [143]. Whether “constricted junction” or other putative barrier mechanisms show selectivity or exist separately for different lipids is an important question still to be answered.

In conclusion, a regulatory system must be in place that controls the asymmetric distribution of lipids between the outer and inner leaflets of the NE membrane, and between the INM and ONM, and the INM and NR. More studies will be needed to understand these challenging questions.

## 5 Where Is Research on Inner Nuclear Lipids Heading?

As discussed in the above sections, there are clear differences between the lipid content of the subdomains of the ER, including the ONM and INM. The different classes of lipids varying in their headgroups have a major impact on the properties of these membrane domains. However, another major factor that affects the biophysical properties of membrane is the fatty acyl side chain composition of the individual lipid species that shows a large variability in length and unsaturation among the lipid classes. These fatty acyl side chain profiles of different lipid classes also vary among different organisms [144]. Currently available methods are unable to generally assess the fatty acyl side chain profiles of the various classes of lipids associated with various organelle membranes. Only organelle fractionation combined with mass spectrometry can provide this information, but cell fractionation has its own caveats introducing a significant uncertainty due to co‐purification of contaminating membranes, including adjacent organelle membranes that form contact sites. Recent studies experimenting with biosensors that could monitor membrane lipid saturation show some promise as they were used to identify mechanisms that protect the INM from excess unsaturated lipids and prevent lipotoxicity during periods of cellular stress and NE repair pathways in yeast [107, 145, 146].

Other areas of interest include questions of how extracellular cues are communicated from the PM to the nucleus through lipid signals as will be outlined below with a few examples.

### 5.1 The Communication Between Cytoplasmic and Nuclear Lipid Signals

As mentioned above, PS synthesis is tightly controlled by feedback inhibition of the PSS1 enzyme by the PS content of the ER [22, 23, 24]. Due to the impact of nonvesicular PS transport between the PM and the ER on ER PS levels, the PS content of the PM has a significant impact on the activity of the PSS1 enzyme [12, 26, 27, 28]. This, together with the finding that the INM PS content closely mirrors the activity of the ER‐localized PSS1 enzyme, suggests that the PS content of the PM could also affect the PS content of the INM. It has also been shown that the activity of the PS transport machinery depends on the PI(4)P gradient between the PM and the ER, and in the case of the ORP8 protein, it is also dependent on the PI(4,5)P_2_ content of the PM [28]. Therefore, changes in PI(4)P or PI(4,5)P_2_ levels in the PM could impact the PS levels in the ER and even in the INM. These are just a few examples of how changes in PM lipids can have a major impact on the lipid composition of the INM. In other forms of regulation, several enzymes responsible for lipid metabolism, such as Lipin1, shuttle between the cytoplasm and nucleus in response to different extracellular stimuli and, hence, alter the ratio of PA to DAG in the INM. Changes of these lipids in the INM then could reprogram the entire metabolic network through control of gene expression. The recently discovered role of the Lem2 in controlling the membrane flow of lipids into and out of the INM is another way of regulating the relative amounts of membrane mass associated with the NE and the ER proper [140]. How this process responds to metabolic demands and environmental cues is an exciting area of future research to explore.

### 5.2 Information Flow Between INM, NR, and Nucleoplasmic Subdomains

It is now evident that the exchange of lipids within the various organelles occurs primarily by (1) vesicular transport and (2) nonvesicular lipid transfer primarily at organelle contact sites [147]. Many lipid transfer proteins that mediate these processes have been identified [25]. At the same time, several studies indicate that nuclear lipids can be exchanged between intranuclear structures and the INM [148, 149, 150, 151, 152, 153, 154]. In particular, it will be interesting to explore whether the NR and the INM have unique lipid properties that determine whether proteins prefer to interact with the membranes of the INM or the NR. Yet another exciting research area is the relationship between nuclear lipids that are not membrane‐associated, such as some of the phosphoinositides, and the intranuclear membranous network. Recent studies have shown that lipid transfer proteins are important to deliver lipids to the nucleoplasmic compartments [155, 156], but where they pick up their lipid cargo and how they release it to nonmembranous intranuclear structures are open questions to be addressed in future studies.

## 6 Concluding Remarks

Significant insights have been gained into the composition and functions of the lipids present in the INM and their enzymology. As is often the case, most of these studies created even more questions than they answered, prompting further inquiries. One of the many fascinating questions is how the various lipids contribute to the NE disassembly at mitotic onset and reassembly after mitosis. At what point does the unique lipid composition of the INM and ONM develop? It is well established that defects in the nuclear envelope lead to the development of a range of human diseases collectively called laminopathies and also contribute to the aging process and tumor formation [157, 158, 159]. What lipid changes are associated with laminopathies, and which ones are secondary or causative in the ensuing functional defects are important questions to investigate. It is safe to say that the lipid biology of the nucleus, including the membranes of the NE, remains a rich area of future research with very high clinical relevance.

## Author Contributions

Yang Niu and Tamas Balla wrote the manuscript. Yang Niu has drafted the figures.

## Conflicts of Interest

The authors declare no conflicts of interest.

## Data Availability

Data sharing is not applicable to this article as no datasets were generated or analyzed during the current study.
